# The University of Buffalo—Meeting of the Council

**Published:** 1883-03

**Authors:** J. N. Matthews

**Affiliations:** Secretary


### The University of Buffalo.

### *Meeting of the Council.*

A meeting of the Council of the University of Buffalo was held at the committee-room of the Young Men's Association, at 4 o'clock, P. M., February 27th. Present, the Hon. O. H. Marshall, Chancellor of the University, in the Chair, Dr. Thomas F. Rochester, Dr. Charles Cary, the Hon. E. G. Spaulding, the Hon. James O. Putnam, the Hon. E. C. Sprague, Mr. J. N. Scatcherd, Mr. John Wilkeson, Mr. J. D. Shepard, Mr. George S. Hazard, and Mr. J. N. Matthews, Secretary.

Dr. Charles Cary read the following report:

We, the undersigned Curators and Faculty of the Medical Department of the University of Buffalo, having duly examined the candidates named below have found them qualified, and do recommend them to the Council to receive the degree of Doctor of Medicine:

GRADUATES.

Herbert Wells Davis, Randolph, N. Y. Pneumonia.

Carlos E. Bowman. Bowmansville, N. Y., Cerebro-Spinal Fever.

Wm. Fletcher Wells, Rushford, N. Y., Acute Peritonitis.

Ephraim Wm. Bogardus, Seneca Falls, N. Y., Pneumonitis.

Ben Gordon Long, Buffalo, N. Y., A peculiar case of Labor, requiring artificial delivery.

Reuben Marion Root, Gaines, N. Y., Pneumonia.

Lucius Gilbert Waterman, Centreville, N. Y., Diphtheria.

Edwin Wilson Baker, Machias, N. Y., Railroad Injuries.

Delos Burd Manchester, Cooperstown, N. Y., Injuries to Veins.

Miles E. Reed, Rome, Pa , Acne.

Byron S. Coleman, Brockpo t, N. Y , Hemorrhoids.

Dewitt Clinton Greene, Buffalo, N. Y., Cerebro-Spinal Meningitis.

John T. Harris, Mitchell, Canada, Cerebro-Spinal Meningitis.

Clark Getman, Columbia, N. Y., Asthma.

Albert Bradley Wilson, Buffalo, N. Y., Acute General Peritonitis.

Burt E. Bidleman, Tunkhannock, Pa , Intermittent Fever.

Samuel Amos Clark, Fredonia, Pa , Tetanus.

Wm. O'Reilley Bradley, Rochester, N. Y., Syphilis.

Wm Oliver Rodgers, Jamestown, Pa., Medical Education.

Peter Eugene Rivard, Rochester, N. Y , Hygiene and general management of health during Pregnancy.

Frederick G. Trowbridge, Watertown, N. Y., Atony and Paralysis of the Bladder.

Major Albert Veeder, Lyons, N. Y., Diseases due to Marsh Miasm.

Frank Robert Burroughs, Corry, Pa , Pertussis.

George Warren Squires, Churchville, N. Y., Spasmodic Asthma.

John Evelen Caneen, Leon, N. Y., Acute Diffuse Nephritis.

Frank Benj. Darling, Leon, N. Y., Acute Articular Rheumatism.

Frederick Ransom Campbell, Rochester, N. Y., The Economic Aspects of Sanitary Science.

Louis Hanly Merchant, Oswego, N. Y., Albuminuria occuring in Pregnancy.

Isabella H. Stanley, Dunkirk, N. Y., Rational Obstetrics

David H. Reed, Remsen, N. Y., Alcohol, and its action and uses.

Samuel S Kilvington, New York, N. Y., Ovariotomy.

Wm. Thomas Wallace, Olean, N. Y., Digestion.

Richard Ward Bamber, Carlton, N. Y., Syphilis.

Hudson G. Willse, Columbia, N. Y., Communicable Diseases and Prevention of Spread.

Charles Cook Ransom, Richfield Springs, N. Y., Median Lithotomy.

John Gurdon Webster, Bath, N. Y., Identification of Human Blood.

Alpheus Prince, Cl.rence, N. Y., Scarlet Fever.

Wm. Harvey Thornton, A. B., Buffalo, N. Y., Bacteria and their relation to Disease.

Samuel Inkermann Wells, Ft. Erie, Canada, Different modes of Dying.

Julius Pohlman, Buffalo, N. Y , An experimental study of the action of Pilocarpine.

Wm. Meisburger, Buffalo, N. Y , Secale Cornutum in Obstetrics.

Myron A. King, Perry Center, N. Y., Senile Constipation.

James E. Crichton, Perry Center, N. Y., Atmosphere.

Albert Wanzer Scofield, Angelica, N. Y., Medical Thesis.

Martin Elihu Gilbert, Yales, N. Y., Pneumonia.

Fred. Wilcox, Bellona, N. Y., Cerebro-Spinal Fever.

John Coulson Parmenter, Buffalo, N. Y., Treatment of some forms of Intestinal Obstruction.

Wm. L. Wooden, Fairport, N. Y., Dyspepsia.

Bina A. Potter, Ithaca, N. Y., Variola.

John Edward Sutton, Albion, N. Y., Cæsarean Section.

James McAuliff, Olean, N. Y., Artificial Anæsthesia.

Wm. Augustus Gray, Tioga, Pa., Pyretics and Anti-pyretics.

Fletcher W. Brockway, Swartwood, N. Y, Signs of Pregnancy.

George F. H. Bartlett, Ph. B., Buffalo, N. Y., Albuminuria.

Albert Walter Cottrel, Whitesville, N Y. Diphtheria.

John Gray, Avon, N. Y., Abortion.

Sophia Jones, Cattaraugus, N. Y., Chronic Constipation.

THOS. F. ROCHESTER, Dean.
CHARLES CARY, Secretary
For the Faculty.

| | |
|---|---|
| William Warren Potter, | John N. Cotes, |
| H. P. Hall, | Jas. W. Craig, |
| A. E. Ellenwood, | Thos. D. Strong, |
| Chas. H. Richmond, | G. H. Lapham, |
| C. B. Kibler, | J. B. Samo, |

W. P. Bemus,

On motion of Dr. Rochester, the persons whose names were thus reported were declared worthy to receive the degree of Doctor of Medicine, and the Chancellor was authorized to confer upon each of them that degree.

Dr. Rochester nominated Dr. M. Picket, of Corry, Pa., as one of the Curators, and he was unanimously elected.

Dr. Cary nominated Dr. Mullen, of Alexander, N. Y., as one of the Curators, and he was unanimously elected.

Dr. Cary then read the following communication:

BELLEVUE HOSPITAL MEDICAL COLLEGE,
New York, March 22, 1882.

*To the Council and Faculty of the University of Buffalo:*

GENTLEMEN—It is with great reluctance and sincere regret that I am compelled to resign my position of Professor of Chemistry at the Medical College.

This course is forced upon me in order that I may accept an appointment in New York at the institution where I obtained my collegiate training. Besides many advantages that will spring from my new sphere of labor, that of being brought into more intimate relations with a devoted father is the most important. Ever since I accepted the Chair of Chemistry at Buffalo, he has been put to great inconvenience owing to my absence, and it is my duty and a great pleasure as well to be able to requite in small measure his self-sacrifice. Having taken upon me matrimonial relations, it is not as easy as it was heretofore for me to lead a sort of a nomadic existence, and I consider it important for me to concentrate my efforts and scientific labors in my own home.

It is painful to sever a connection which has brought me so much keen enjoyment. My relations with the Faculty and Council have always been so agreeable that I shall have only pleasant memories of one and all.

Going as a stranger among you, I appreciated the kindly welcome extended from all sides, and though the occasions of our meeting in future may be fewer, I shall hail them with pleasure.

During five years' labor in a common cause I need not assure my colleagues that my interest in the success of the school will not terminate with that of my official capacity as an instructor.

I am indebted to my associates for a most cordial seconding to all suggestions I offered concerning the department of chemistry. Especially was this the case with our respected and beloved President.

Thanking you for the mark of confidence which you manifested when you called me to so honorable a position, and trusting that I may continue in your esteem, I have the honor to be, Your obedient servant,

CHARLES A. DOREMUS.

On motion of Dr. Cary, the resignation of Dr. Doremus was accepted.

Dr. Rochester moved that Dr. R. A. Witthaus, of New York, be elected Professor of Chemistry and Toxicology, and he was unanimously elected.

Dr. Cary then read the following communication, which he said was written in reply to a letter from the Faculty, appealing to Professor Miner to retain his Chair:

BUFFALO, Sept. 26, 1882.

*To the Faculty of the Buffalo Medical College, through Professor Charles Cary, M. D., Dean of the Faculty:*

The communication from Professor Stoddard informing me of the action of the Faculty at your late meeting is received and contents noted.

Feeling confident that my action in presenting my resignation is well-advised, being the result of careful consideration in view of all the circumstances, I see no reason for changing my mind, and desire you to regard it as final.

I assure you I entertain none but the most kindly feelings toward my colleagues, and fully appreciate their many acts of kindness extended to me during my recent illness, and for which I desire they will accept my sincere thanks.

I sever my connection with your honorable body with best wishes for the prosperity of the College, and trust the very pleasant relations I have enjoyed with the Faculty may not be interrupted or diminished.

Yours, very respectfully,

JULIUS F. MINER.

Dr. Cary said that the Faculty had felt constrained to accept this resignation, and they recommended that Dr. Miner be appointed Emeritus Professor of Special Surgery.

On motion of Dr. Rochester, Dr. Miner was unanimously elected Emeritus Professor of Special Surgery.

Dr. Rochester, in behalf of the Faculty, extended a cordial invitation to members of the Council to attend the banquet at The Genesee this evening.

The Chancellor suggested that it might be convenient to the Council to have a President other than the Chancellor. He therefore offered his resignation as President of the Council. The suggestion was not acted upon.

On motion of Dr. Rochester, the Secretary of the Faculty was instructed to write to Dr. Austin Flint, of New York, requesting for publication a copy of the address delivered by that gentleman at the last commencement exercises.

On motion the Council adjourned.

J. N. MATTHEWS, *Secretary.*

